# Analysis of the application of artificial intelligence technology in the protection of corporate governance rights and interests

**DOI:** 10.3389/fpsyg.2022.966689

**Published:** 2022-09-12

**Authors:** Wenjun Shen

**Affiliations:** Law School, Xi’an University of Finance and Economics, Xi’an, Shaanxi, China

**Keywords:** artificial intelligence, corporate governance, decision-making, management control, policy regulation

## Abstract

Corporate governance delivers feasible and controlled company operations using a group of common shareholders and appropriate policies. The roles and responsibilities of the shareholders suggest and improve corporate development through monotonous and independent rights. The implication of artificial intelligence provides knowledgeable insights for decision-making and control management. This article introduces a Mutual Consent-based Governance Regulation Model (MCGRM) for dissimilarity mitigation in corporate rule implications. The proposed model exploits transfer learning for balanced rule implication and decision-making. The learning states are defined based on mutual agreement, individual interest, and operational features. Based on the governance policies, the above rules are employed without hindering the pioneer regulations implemented in different periods. Therefore, artificial intelligence technology is utilized for prompt and swift governance decisions in delivering special rights for consumers and shareholders. The performance of this model is validated and verified using data sources related to governance policies from a real-time industry. The impact of varying policy features with dissimilarity is analyzed for varying occurrences. The analysis is given based on the considered data sources for which the classification and its impact over reports, sharing, voting, complaint, and market are analyzed. The availability before and after the proposed improves the above metrics by 10.48, 10.65, 9.78, 13.39, and 9.26%.

## Introduction

Corporate governance is a system or technique that provides various directions, rules, policies, and controls over companies and organizations. Corporate governance includes security and safety measures that the Board of directors issues in companies (Caixe, 2021). Corporate governance maintains and manages control such as human rights aspects, transparency in providing salaries, and the company’s carbon footprints. The primary purpose of governance is to provide a better decision-making process and improve a company’s accountability rate (Orihara and Eshraghi, 2022). A corporate governance system improves the positivity rate in behaviors, reduces computation cost, improves decision-making accuracy rate, and provides strategic plans. Corporate governance protection is a process that offers a secured set of policies for the users to protect the investors and shareholders via large suits (Min et al., 2022). Corporate governance protection handles the laws by providing the exact meaning and understanding over law firms. Corporate protection provides evidence for improving firm-level, which improves companies’ overall performance and effectiveness. Governance provides policies for shareholders and investors to ensure the safety and security of personal details from third-party members (Cosma et al., 2018; Cheffi and Abdennadher, 2019).

Artificial Intelligence (AI) techniques are widely used in various fields to provide better and improved user services and increase an application’s feasibility and efficiency. Corporate governance uses AI techniques to facilitate customer and shareholder interests in the organization (Bonsón et al., 2021). The primary role of AI is to perform the tasks with autonomous, efficient, augmented, and amplified scenarios, which eliminate unnecessary fears and threats to the governance system. AI provides relevant activities for every actor inside a corporation (Sun, 2021). Actors such as workers, managers, investors, shareholders, advisors, and customers play significant roles in improving an organization’s performance. AI gathers every actor’s job and forms a sequence to perform a particular task (Di Vaio et al., 2022). AI-based applications are commonly used in various companies to provide accurate corporate governance policies and improve the security level of customers from attackers. Internet of Things (IoT) based applications are also used in the corporate governance system, which issues an actual set of law firms and conditions for users and provides necessary services to avoid unwanted threats in the investment process. AI finds out the real potentials of companies and tries to offer optimal solutions to solve the problems in the corporate governance system. AI also identifies the unstable business proposals and unstable strategies in the organizational environment, reducing companies’ failure rates (de Almeida et al., 2021; Lipai et al., 2021).

Machine Learning (ML) techniques are widely used for detection and analysis processes, providing an accurate set of details for further processes. ML techniques are used in corporate governance systems to improve their decision-making ability and reliability (Stahl et al., 2022). ML-based reliability model is used in the corporate governance protection process, providing transparent operation and function for both users and investors. Reliability theory is used here to find out the behavior and strategies of companies and produce a final set of data for the analysis process (Van Dijk et al., 2021). Both processing and failed operations are identified using the ML technique, which reduces the error rate in the governance protection process. A structure-function is used in the reliability model to detect critical components and sources in qualifying services (Villarón-Peramato et al., 2018). The auditor choice prediction model is used in the corporate governance protection system, which prevents both customers and investors from third parties. The convolutions neural network (CNN) approach is used in the prediction model, which predicts the weak strategies and conditions in the financial process. The auditor choice dataset is stored in the database, which improves the overall accuracy rate in the prediction process and provides a relevant set of data for the analysis process (Ahmad et al., 2021; Miglionico, 2022). The contributions of this article are listed below:

i Designing a Mutual Consent-based Governance Model for improving the support for decision-making in corporate governance
ii Performing a classification-based validation by considering standard data sources for validating the proposed model’s performance
iii Assessing the independent impact of the features associated with the data set for improving the reliability based on fundamental availability rights to the shareholders and regulations

Therefore, the article organization is as follows: In see section “Related works,” the related works from different authors with the functions, working, pros and cons are discussed. In see section “Proposed mutual consent-based governance regulation model,” the proposed model with the proposed implication and suitable illustrations are examined. See section “Discussion” presents the analysis of the proposed model’s performance through data analysis, and the article is concluded with the future scope in see section “Conclusion.”

## Related works

Wang et al. (2022) introduced a configuration analysis of the corporate social responsibility (CSR) method for increasing the financial performance of corporate companies using the Quantitative comparative analysis (QCA) approach. The first configuration process includes highly responsible governance, interests, and employee rights. Public charity and effective environmental protection are included in the following two configurations. The proposed method improves the overall performance and efficiency in providing user services.

Jun et al. (2021) proposed a hybrid recommendation model for research and development (R&D) collaboration in small and medium-sized enterprises (SMEs). Discriminant analysis and mixing machine learning techniques are used in the proposed model to perform the evaluation process and produce finalized set of data for R&D. The hybrid recommendation model is mainly used to identify the SME and increases the satisfaction rate in performing tasks. The proposed recommendation model increases the accuracy rate in achieving services for users and improves the efficiency rate in the public investment process.

Liang et al. (2020) introduced a bankruptcy prediction model using a stacking ensemble for corporate governance indicators (CGI). A financial ratio (FR) is used in the proposed model to find out the corporate bankruptcy rate. The discriminant analysis method plays a significant role in ensuring CGI’s specification and classification process. Compared with other traditional methods, the proposed prediction model increases the prediction accuracy rate and improves the governance system’s performance rate.

Kim (2020) proposed a new detection method for a principal-agent problem using the deep learning (DL) approach. The proposed method finds out the exact cause of agency relationships, informational asymmetry, and misconduct related to principal-agent problems. DL approach increases the accuracy rate in the detection process, which eliminates irrelevant data from the database. The proposed detection method improves the feasibility and robustness of the corporate governance system, reducing the detection process’s latency rate.

Chang et al. (2020) introduced a qualitative approach for identifying risk factors in the Internet of Things (IoT) governance system. The proposed method influences the governance system finds the internal control in IoT, and then produces an optimal data set for the analysis process. The proposed approach identifies the risk factors presented in enterprise internal control using the classification process. Experimental results show that the proposed approach improves internal control and auditing effectiveness in the IoT environment.

Zhang et al. (2021) proposed a hybrid approach for risk analysis in an e-business environment. The artificial intelligence (AI) technique is used in the proposed approach to finding out the integrity rate in e-business. AI creates a qualified set of financial data used in the risk analysis process and provides the necessary data at a time. The proposed method eliminated the vulnerabilities and fake data from internal storage. The proposed hybrid approach reduces the risk factors and conditions in e-business, improving the system’s reliability.

Robinson (2020) introduced a machine learning (ML) approach for measuring fundamental ratios. Environmental, social, and governance (ESG) scores are used in the proposed approach as a predictor, which provides related data for the analysis process. ESG scores provide data such as structural data and income statements of trade companies. A random forest algorithm is used in the proposed method to find out the actual structural variables for the investigation process. The proposed approach improves the performance and efficiency level of the governance system.

D’Amato et al. (2021) proposed a structuring information security (IS) framework for a corporate system using statistical survey analytics. The information technology department maintains and stores corporate information, ensuring users’ safety from third-party members. The proposed framework increases the feasibility and security level of IS management system using statistical analytics. The proposed framework reduces the error rate in storage, improving corporate companies’ overall efficiency and operation.

Robinson (2020) introduced a sustainable strategy model based on sentiment analysis for the corporate governance system. Corporate social responsibility (CSR) is used in the proposed model to determine the financial performance index by combining it with the non-financial index. Experimental results show that the proposed model improves the accuracy rate in detecting CSR, increasing the corporate governance system’s significance and financial performance.

Song et al. (2018) proposed a diversified investment strategy to find the relationship between the internal capital market and corporate governance. The proposed method identifies the relationship between capital markets and produces relevant data for analysis. Relations such as chairman, ownership, and directors are identified and analyzed using certain features. The internal capital market plays a significant role in diversified investment strategy, improving users’ investment rates.

Cui et al. (2019) introduced a reliability model using machine learning (ML) techniques for assessing corporate governance. The mapping approach is used here to determine the characteristics and functionalities of an internal capital interest in corporate governance. The proposed method is commonly used in non-financial and start-up companies to improve the feasibility and reputation among the users. The proposed reliability model reduces risk factors that will enhance governance system performance.

Hernandez-Perdomo et al. (2019) proposed a new annual financial statement analysis process using machine learning (ML) models for corporate governance systems. The proposed method is used to identify the financial statements and risk factors presented in the governance system. ML identifies the patterns which provide related data for detecting characteristics. Experimental results show that the proposed method improves the feasibility and scalability of the corporate governance system.

Wyrobek (2020) introduced the corporate accounting information disclosure method using a field-programmable gate array (FPGA) and neural network (NN) algorithm. The proposed method determines the difference between authorized and unauthorized persons in the accounting process. NN prevents the users by denying access control over users’ personal information and accounts. The encryption process plays a significant role in decoding the texts and converting them into meaningful content, which is used for accessing the process. The proposed method improves the performance of the corporate governance system.

Zhang (2021) proposed a prediction process for environmental controversies and development in a corporate environment. The proposed method is mainly used to increase the corporate ecological performance (CEP) rating, which improves the effectiveness of the corporate governance environment. ML approach reduces latency rate and energy consumption rate invalidating the process. The proposed method increases the accuracy rate in improving the performance rating and also predicts the validity of the user’s account.

Svanberg et al. (2022) introduced personal executive characteristics and corporate performance governance using the machine learning (ML) approach. The proposed strategy is used to find out the relationship between features and performance. The proposed method uses the Boosting regression tree algorithm to investigate the characteristics and avoid weak information from the queue. The proposed strategy increases the overall performance and effectiveness of the corporate governance system.

Yang et al. (2022) analyze the corporate life cycles to predict the changes in bankruptcy and earning management. Corporate life cycle, different stages are investigated to manage the business earnings. This work uses hierarchical mixed methods to explore multi-level data for improving business earnings. The author uses the 33,000 central European companies’ information to investigate the financial indicators and enhance the profit quality.

Durana et al. (2021) investigated Sri Lanka’s corporate governance to manage the company structure. The main intention of this study is to explore whether corporate governance affects the company’s decisions or not. The author uses the 138 non-financial companies collected from 2009 to 2013. During the analysis, board committee, leadership structure, board composition, managerial information and board size details are utilized to investigate the return on assets. The collected details are processed by applying the multiple regression analysis to identify the relationship between governance and company profit. The above-discussed methods rely on multi-factor or stagnant features for analyzing the governance model. This improves the sustainability of restricted shares and operations wherein the components are retarded due to multi-dimensional market flow and demands. Therefore, a mutual and amendable rule implication is necessary for improving the internal governance for the varying market risks. Thus, this article introduced a mutual consent-based governance regulation model for suppressing the stagnancy in governance rule modifications.

## Proposed mutual consent-based governance regulation model

**FIGURE 1:**
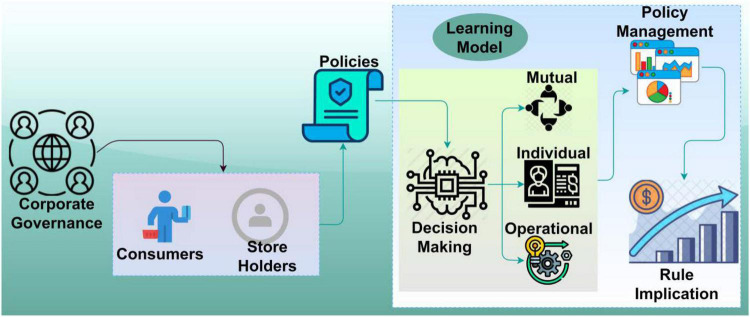
Proposed model.

This model discusses the protection mechanisms and decision-making of corporate governance policy management control and rule implication. Economists have used the idea to model appropriate financial instruments regarding the rules and regulations they provide to their consumers and shareholders. The shareholders notice the consumer problems because they have standard rules, an interest in maximizing profit, and enough control over the firm’s policies to respect their interests. The proposed model is featured in Figure 1.

The proposed MCGRM model is designed to analyze the protection and responsibilities of corporate governance rights and interests to ensure prompt and swift governance decisions in delivering special rights for consumers and shareholders using better artificial intelligence technology utilization. The policies defined for corporate governance rely on decision-making for individual, operational, and mutual agreements. This is validated under standard guidelines for improving the amendments and meeting the market requisites. The balancing factors such as internal (consumers) and external (shareholders) rules and regulations are held by policies through monotonous and independent rights in a consecutive manner. It ensures the decision-making and control management solution from different knowledge insights. The rule implication and decision-making process differ from consumers, and its dissimilarity mitigation is balanced using transfer learning. Individual interest and operational features are jointly analyzed through policy changes in a learning state based on mutual agreement. Therefore, this policy modification is responsible for implementing the pioneer regulations in different periods delivering less precise rights for consumers and shareholders. The rule implication is modeled for the existing policies and decision-making. The decision-making is feasible to be employed for policy management within the same corporate governance (Refer to Figure 1). This method aims to maximize feasible deliveries and control company operations for corporate governance rights and interests. The accumulative and probabilistic corporate governance delivers, and company operations results in prompt and swift governance decisions of reducing policies considered consumers and shareholders, respectively, then


(1) $$\sum_{i=A}{\left(C,S\right)}_{i}=\sum_{i=A}\sum_{p=t}{\left(C\right)}_{i\,p}-1-\left[\frac{{\left(C\right)}_{i}}{\sum {\left(C+S\right)}_{p}}\right]$$

In Eq. 1, the variables *C* and *S* are used to represent the consumer and shareholders through Artificial Intelligence Technology *A* in the corporate governance *c^g^*. The maximum probability of *M*_*x*_ = 1 achieves high *C* and *S* for the corporate governance operation. If *i* denotes the number of processing instances whereas *p* represents the different periods. Therefore, the variables *A* and *t* are not idle due to the protection of corporate governance as *M*_*x*_ ∈ [0,1] is the varying constraint. Instead, *M*_*x*_ = 1 is not assured at different periods *p*, resulting in dissimilarity. This issue is referred to as policy management in a corporate governance analysis and controlled company operations scenario using a group of ordinary shareholders and appropriate policies. Artificial Intelligence Technology and the learning model are jointly used in the proposed corporate governance of maximum utilization.

### Proposed model for corporate governance policies

In an Artificial Intelligence Technology uses Governance Policies, the shareholders’ operations are achieved by modifying policies from the corporate governance rights and interests. The shareholders’ rules and responsibilities suggest that monotonous and independent rights take place in corporate development through Artificial Intelligence Technology. The corporate governance policies are classified on the below-represented features.

**FIGURE 2:**
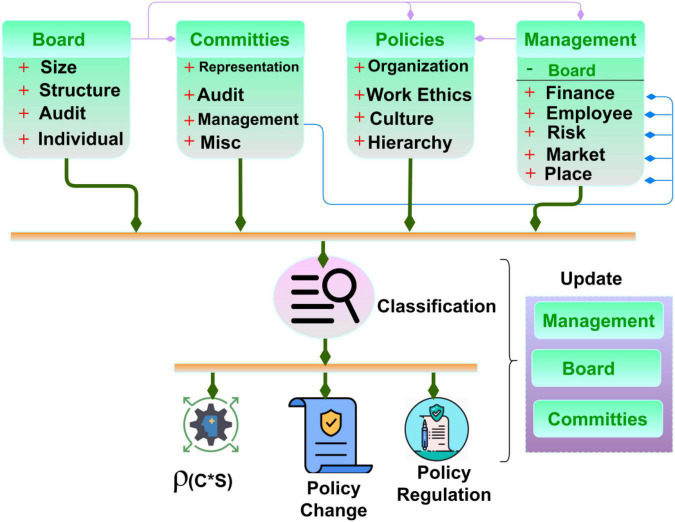
Governance policies classification.

A sample of the governance policies based on different organizational representations is presented in Figure 2. The above classification is a general representation of the limited features available in a corporate governance structure. This is modified for understandability, as illustrated above, with the appropriate notation illustrations. The classification observes changes in different levels [Board, committees, policies, and management (say)] from whichρ(*C*×*S*) and $P_{L}^{R}$ are determined. This is required to achieve fair *S_p_* such that *C_i_* and *C_p_* are reflected. The governance policies consist of consumers and shareholders to identify the contrary occurrence of the dissimilarity mitigation, as in the above Eq. 1. The probability of consumer to shareholder considered policies in different *p* without changes (i.e.) ρ(C*S) is given by


(2) $$\rho \,\left(C*S\right)=\frac{\sum_{i\in p}c\,h_{i}}{\sum_{i\in A}c\,h_{p}}\,P_{L}^{-\frac{c\,h}{A}.\exists .i}$$

Where the variables *C*_*i*_ and *C*_*p*_ represents the policies changes analysis of the *c^g^* in a given time *p* and the appropriate policies *P_L_*, respectively. For instance, the policy changes the expression of $1-\left[\frac{P_{L}\,{\left(c\,h\right)}_{i}}{\sum {\left(C+S\right)}_{i}}\right]$ is calculated using∃. The first condition for maximizing ρ(*C*) is *M*_*x*_ = 1 as the differing factor; therefore, this is the policy-changing metrics, and therefore decision-making is performed based on the *C_i_*, *n* ∈ *t*. The corporate governance using *A* in *p* helps to compute the output for both consumers and shareholders *P_L_*. This policy change is identified using Eq. 3 and is valid in *p* alone.


(3) $$ch_{p}\forall i\in p=[\left(1-\exists \right)\frac{C}{c\,h_{i}}.M_{x}.\frac{C}{c^{g}}-\left(ch_{i}-ch_{i}^{\exists }\right)i\in A$$

The above Eq. 3, the policy changes analysis in the available *c^g^* is identified and provides knowledge about previous policies in *p*. Therefore, in this condition *ch*_*p*_∀*n* ∈ *t* exploits, then decision-making is required. A failure in policy changes maximizes∃, defacing the delivers and company operations of corporate governance. The policy rules and regulation hold the available *C* and position of *A* as {*M_x_*, *ch*,*ch*^∃^,ρ(*C*)} after the policy changes or *S* in different periods *p*. The output for the dissimilarity mitigation in corporate development of *S* and *ch*_*i*_∀*i* ∈ *A* is analyzed through Artificial Intelligence Technology and is provided knowledgeable insights into management control. The outputs are accounted for as further processing of decision-making and rule implication. In this manuscript, the policy changes are identified and proceed using the learning process, and it depends on *ch* and *M*_*x*_ for $\sum_{i\in A}{\left(C\right)}_{i}=C$ and *ch*^∃^ and ρ(*C*) for the varying condition in the above Eq. 1. Let *S*_*p*_ and *S*_∃_ denote the shareholders’ policy validity periods and further computation of *C* in both conditions in the above-derived equation. It refers to the consumers’ and shareholders’ policy changes for the corporate governance-based rights and interests. Therefore, the policy regulation $\left(P_{L}^{R}\right)$ is estimated as


(4) $$P_{L}^{R}=S_{p}+S_{\exists }$$

Instead, the above Eq. 4 is substituted in consumer and shareholders’ policies for feasible delivery and controlled company operations, respectively.


(5a) $$S_{p}=\sum_{i\in A}{\left(C\right)}_{i}=M_{x}\times \sum_{n\in B}\frac{{\left(C\right)}_{p}}{c\,h_{i}}=M_{x}\times \sum_{i\in B}c\,h_{p}$$

Similarly,


(5b) $$S_{\exists }=\sum_{i\in A}\sum_{i\in p}{\left(C\right)}_{i\,p}-\left(1-\exists_{i\,p}\right)=\sum_{i\in A}\left(c\,h_{i}-c\,h_{i}^{\exists }\right)\,{\left(C\right)}_{p}$$

**FIGURE 3:**
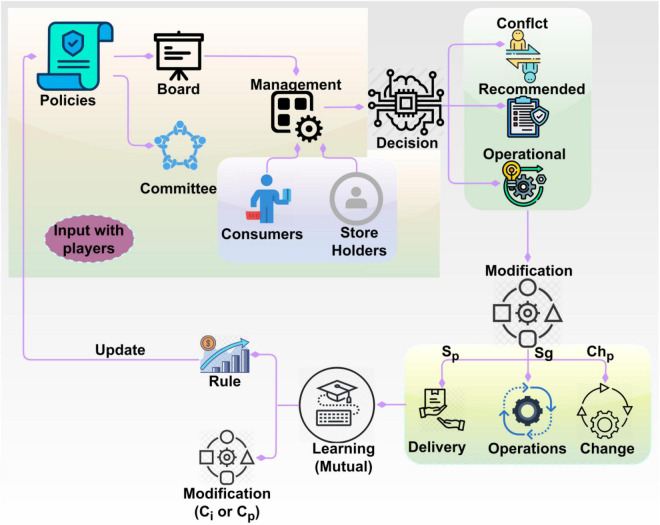
Policy management using transfer learning.

Based on the Eqs. 4, 5a, 5b, $P_{L}^{R}$ is computed as a factor of *C* and *ch* ∃ to calculate the feasible delivers. Therefore, *S*_*p*_ depends on policy changes and *M*_*x*_ whereas *S*_∃_ depends on *S*_∃_ and ρ(*C*). The condition of *S*_∃_ and *M*_*x*_ need not be one, but not zero through implications of artificial intelligence provided knowledgeable insights for monotonous and independent rights can be satisfied successfully. Therefore, the condition of $P_{L}^{R}=S_{p}$, then shareholders suggest and improve corporate development does not occur, the policy changes in corporate governance based on above Eq. 1 do not proceed for further process. This policy regulation relies on *M*_*x*_ = 1 and *ch*_*p*_∀*i* ∈ *p* = *ch*_*i*_∀*i* ∈ *A* conditions for which the knowledgeable insights for corporate governance to the implications of different *p*. The dissimilarity mitigation of 0 < *M*_*x*_ < 1 is acute for the decision-making of $P_{L}^{R}=S_{\exists }+S_{p}$ that is controlled as a different policy period of $P_{L}^{R}\,\left(p\right)=S_{p}\,\left(p-\frac{c\,h_{i}}{c\,h_{p}}\right)+S_{\exists }\,\left(p\right)\,\forall i\in p$ and *i* ∈ *p*, respectively. The policy management (modification) based on transfer learning is presented in Figure 3.

The transfer learning operates on *S_p_*, *S*_*g*_ and *ch*_*p*_ they were observed from the decision outcomes. The decisions are considered from the different classifications (mutual individual and operational). In this output, the consumers/store holders’ impact is considered [input with the player (Refer to Figure 3)]. This learning extends knowledge on(*C*_*i*_*orC*_*p*_) modifications for $P_{L}^{R}$. The regulations (updates) are provided from the accepted (final) rule defined by the learning. However, the *C_i_* or*C_p_* requirements are used for *ch*_*p*_ validation through the decision outputs. The balanced rule implication and decision-making process in the above operation exploits transfer learning for analyzing the policy changes in $\left(p-\frac{c\,h_{i}}{c\,h_{p}}\right)$ as this is the dissimilarity in *p* instance. Where the condition*ch*_*i*_≠*ch*_*p*_at the initial stage of *S*_*p*_ and ρ(*C*) is calculated through $S_{p}\,\left(p-\frac{c\,h_{i}}{c\,h_{p}}\right)$ based on*M_x_*. These two processes are modeled using *ch* metric as


$$P_{L}^{R}=M_{x}*{\left[\frac{\left(\frac{\min{\left(C\right)}_{i}}{\max{\left(C\right)}_{i}}\right)}{C+S}\right]}^{\exists }\,\left(p-\frac{c\,h_{i}}{c\,h_{p}}\right)$$

(6) $$+\frac{\max{\left(C\right)}_{i}-\min{\left(C\right)}_{i}}{\max{\left(C\right)}_{i}}.\rho \,\left(C\right)$$

From the above Eq. 6, the first shareholder policy provides a solution of 1 as *M_x_* = 1,*min* (*C*)_*i*_ = *max* (*C*)_*i*_ and *S* = 0, and*C* = *min*⁡(*C*)*ormax*⁡(*C*). This is different from the cumulative estimation performed in Eq. 4. The impacting*ch*_*i*_ and *ch*_*p*_ are employed for assessing the above over varying(*C* + *S*). Therefore, it is considered as $P_{L}^{R}=\sum_{i\in p}{\left(C\right)}_{i}$ or *C* until $\left[1<p-\frac{c\,h_{i}}{c\,h_{p}}<p\right]$ is accounted. The evidence suggests that corporate managers resist takeovers to protect their private benefits of controlled company operations rather than to serve shareholders. Therefore, the policy powers of each state are available and aid in enforcing the process of contracts or enforcing the collection of damages for non-process. In particular, the consecutive dissimilarity analysis incorporates rule implication of *C* to *S* policies are processed between $\left[p-\frac{c\,h_{i}}{c\,h_{p}},p\right]$ where the probabilistic decision-making and control management is explained in a detailed manner.

### Control management

This subsection discusses the control management over the decision-making for rule implications. Control management considers the impact of mutual, individual, and operational rules. Based on the considerations, the implication is pursued; hence, the learning induces the control process. This control process relies on varying implications and the dissimilarities observed. The previous knowledgeable insights on probabilistic factors *M*_*x*_ andρ(*C*) Influences the balanced rule implication and decision-making factors in corporate governance. Instead, the differing constraints of the above Eq. 1 are analyzed in corporate development to increase rules and regulations. The probability of *S* decision-making is computed as in the following Eq. 7


(7) $$\rho \,\left(S\right)=\frac{\rho \,\left(C\,\cup S\right)}{\rho \,\left(S\right)}$$

The decision-making for corporate governance policies using artificial intelligence technology depends on transfer learning. This learning is used for making a decision based on mutual agreement, individual interest, and operational feature instances. In mutual understanding, the corporate governance principles are accountability, fairness, transparency, responsibility, and rules that are similar for policies and periods. In an individual interest, corporate governance balances the interest of different stakeholders such as customers, the government, shareholders, financiers, suppliers, senior management executives, and the community for policies. Instead, operational features such as effective risk management, clear organizational strategy, fairness to consumers and employees, transparency and data sharing, discipline, and commitment in which the instance is observed and analyzed the emergencies in consecutive policies. Therefore, the above conditions are defined with the governance policies using learning states. The decision-making process depends on changing policies for the corporate governance delivers probabilities while implementing pioneer regulations. The organization’s pioneer regulations are framed and implemented for a successful operation. The above rules are framed based on the modifications and improvements from the shareholders, market values, and other enhancements. Therefore, the conditions for implemented pioneer regulations vary, which follow additional rules, interests, and features through decision-making. The decision-making is based on the consumer and shareholders’ policies by calculating the *S* for available regulations and policies of consumers and shareholders at different periods. The decision-making *D^M^*(*c^g^*,*p*) relies on maximum policies (*p*) and *C* as


$$D^{M}\,\left(c^{g},p\right)=\left[S-\left(\frac{C}{c\,h_{i}-c\,h_{i}^{\exists }}\right)\times \frac{1}{M_{x}}\right]$$

(8) $$-P\,i\,o\,n\,e\,e\,r\,r\,e\,g\,u\,l\,a\,t\,i\,o\,n\,s\,\left(M_{x}\right)+1$$

In these pioneer regulations and further responsibilities computing probability, the aim is to balance decision-making and rule implication to reduce the risk management and therefore, the actual policy management for *C* is given as


(9) $$M_{x}\,\left(C\right)=\max\left[\frac{S_{p}\times \frac{c\,h_{i}}{c\,h_{p}}}{P\,i\,o\,n\,e\,e\,r\,r\,e\,g\,u\,l\,a\,t\,i\,o\,n\,s\,\left(M_{x}\right)-S_{\exists }\,\left(p\right)*\frac{c\,h_{i}}{c\,h_{p}}}\right]$$

Based on Eq. 9, the policy periods rely on rule implication (as per the policy management) is either of *M*_*x*_ (or)*C*, in both the constraints, if*Pioneerregulations*(*M*_*x*_) = 0, then *M*_*x*_ = *C* = *S* is the maximum possible condition, and if*Pioneerregulations*(*M*_*x*_) = 1, *C* = *S*−*M*_*x*_ or*C* = *S*. Hence, the occurrence of *S* = *C* is a feasible delivery where the precise rights for all the consumers and shareholders (without hindering the implemented pioneer regulations) are given in Eq. 1. This is appropriate for all *i* ∈ *p* and *i* ∈ *A* in the Eq. 1. In Eq. 8, the decision-making for mutual, independent, and operational features is analyzed to provide unanimous feature updates for different considerations. Equation 9 presents the acceptable (joint) policy feasible for the implication to ensure maximum data availability over the varying input features. This further covers the entire organizational governance. The artificial intelligence technology is utilized in this scenario for prompt and swift governance decisions available to all shareholders at different periods, where consumers and shareholders are balancing, and therefore the policies are compact as in Eq. 1. The assessment of $\rho \,\left(P_{L}^{R}\right)$ depends on the contrary part of the above-derived Eq. 1 and in the ρ(*C*) is the part of the above Eq. 6. In any instance of*S*, if*C* < *S*_*p*_, then dissimilarity takes place, which again results in decision-making. The rule implication *R*_*Im*_ for both ρ(*S*) and ρ(*C*) is computed in the sequential occurrence of *P*_*L*_ to ensure *C* > *S* as in Eq. 10


(10) $$R_{I\,m}\,\left[\rho \,\left(C|S\right)\right]=\frac{1}{\exists }\,e\,x\,p\,r\,e\,s\,s\,i\,o\,n\,\left[\frac{{\left(C-\exists \right)}_{i}^{2}}{2\,\exists }\right],\forall i\in A\,i\,n\,p$$

**FIGURE 4:**
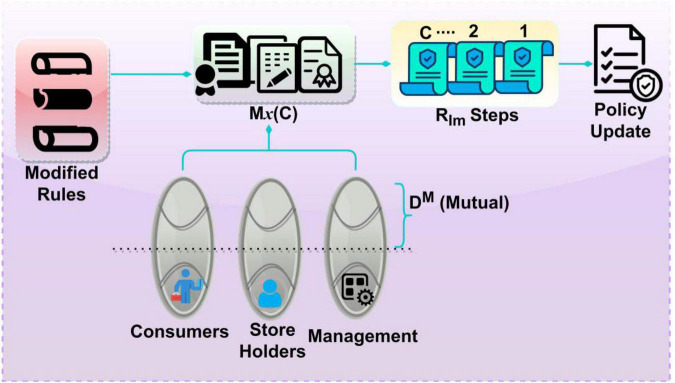
Policy management from decision-making and rule implication.

The consideration of policy regulations as a controlled operation of *p* and $\left[p-\frac{c\,h_{i}}{c\,h_{p}}\right]$ Instances, the above probability of rule implication is estimated based on*p*. Therefore, the occurrence of either of the instances utilizing *A* in any*p* is considerable for increasing $P_{L}^{R}$. The above *R*_*Im*_[.] based on ρ(*S*) andρ(*C*). Figure 4 portrays the policy management steps from decision-making and rule implication.

The modified rules for *M_x_* (*C*) is implemented using*R*_*Im*_ throughout*C*. This is required for*D^M^* for different shareholders, management, and consumers. The mutual impact over the varying features is analyzed post the learning iterations. Therefore, the policy update is based on maximum features for analysis, and hence the*Max*(*C*) is incorporated for amending different policies. The policy management and update rely on *R* ∈ ρ(*S*)∀(*C^g^p*). This is achieved from the learning process using *S_p_*, *S*_∃_, and*ch*_*p*_ features. Based on this, the modified rules are verified for their adaptability with the consumers, shareholders, and management. If *D^M^* (*c^g^*, *p*)is expected, then*R*_*Im*_ is implemented eventually. This verifies *S^p^* without hindering $P_{L}^{R}$. The assessment of rule implication *R*_*Im*_[.] for all the instances, the policy management differentiates the decision-making of $\left(S_{p}^{1}\,t\,o\,S_{p}^{\exists -1}\right)$ for policy regulations from*C*. The consumer and a group of shareholders are available in all *p* during the control management process. Therefore, the pre-decision making based on knowledge other than*ch*, the first period of [1,∃] assists in analyzing *p*∀*C* to improve the policy management of corporate governance in 0 < *M*_*x*_ < 1 condition for any *S* with different *c^g^*. The proposed model balances ρ(*S*) > ρ(*C*) until time split is calculated where different *C* is provided independently for implicating the rules based on governance policies. In this condition, if the consumers and shareholders increase, the minimum dissimilarity mitigation is attuned. Therefore, the rule implication and decision-making are consecutively improved through the learning model, which identifies the rights in that corporate governance through artificial intelligence technology.

## Discussion

In the discussion section, the real-time representations from NRGMetrics (2016), Bulathsinhalage and Pathirawasam (2017), and Mansour et al. (2020) are used. The representations in the second dataset are classified as recommended in the first (Bulathsinhalage and Pathirawasam, 2017) for further analysis. The first dataset (Bulathsinhalage and Pathirawasam, 2017) provides the variables, features, organization, and representation of corporate governance structure. The dataset consists of Board of director related key variables; Audit related three key committees, nomination and compensation information. In addition, 60 variables are presented respected to corporate governance. In the second dataset (NRGMetrics, 2016), 17 firm segments are classified as transparency, Board, and shareholder rights. Each classification is represented by 15, 9, and 8 rules. This dataset consists of 570 observations collected from 95 non-financial firms’ information. The information is gathered from the securities depository center. In addition, the 49 industrial sector firms are compiled from nine segments. Based on the rules, analysis for classification and policy management sources are discussed below. The research for hotels and tourism firms from NRGMetrics (2016) is used for the above analysis. The third dataset utilized in this work is Sri Lanka company information collected from 2009 to 2013. The dataset consists of Colombo stock exchange information gathered from 138 companies. The dataset covers 17 sectors with variables such as Debt ratio, the board size, board composition, CEO duality, board committees, managerial ownership, return on assets and firm size.

### Analysis of classification

**FIGURE 5:**
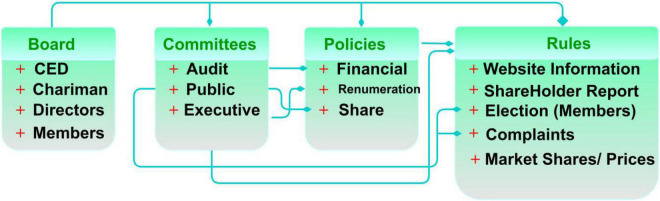
Classification of “hotel and tourism” industry.

**FIGURE 6:**
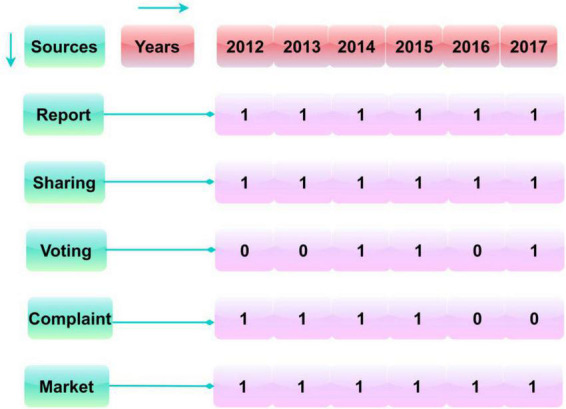
Data generator (source) representation.

The classification for the hotel and tourism industry is presented in Figure 5. This classification is based on the representations in Yang et al. (2022) and the data generators in Durana et al. (2021). The data generators (source) for this industry are diagrammatically represented in Figure 6.

From the information (Durana et al., 2021), the above classification for “hotel and tourism” is represented in Figure 5. This classification is considered a single area due to variations observed in this field. The data availability is less, and the variations are presented in Figure 6 based on which the validation is performed. This would improve the proposed method’s efficiency. In the future perspectives, limited data-based analyses will be performed based on further optimizations. A few relationships between the classifications are highlighted in the above representation. The relies based on reports, sharing, voting, complaint, and market prices are analyzed and modified using the proposed model. Based on the above, the source (data) is classified.

In Figure 6, the availability is marked as one, and the unavailability is marked as 0. This representation is for nine observations under “hotel and tourism.” Based on the above, the before and after implications of MCGRM and its impact on the five sources are analyzed. The policy management sources are diagnosed with the before and after effects of MCGRM implication is discussed below. The impact analysis analyses the before and after application of the proposed model over the considered dataset. This refers to the data analysis in a conventional manner and the validation post the MCGRM implication for analyzing the availability based on the above representation. The dissimilarity measure is also considered for the varying observations in the analysis. The dissimilarity measure is estimated as the variation between individual, mutual, and operation decisions made over different*p*. This is uncertain based on the existing and recommended policies from the learning decisions. The proposed model identifies these observations between availability and unavailability as a dissimilarity measure.

#### Impact on reports

**FIGURE 7:**
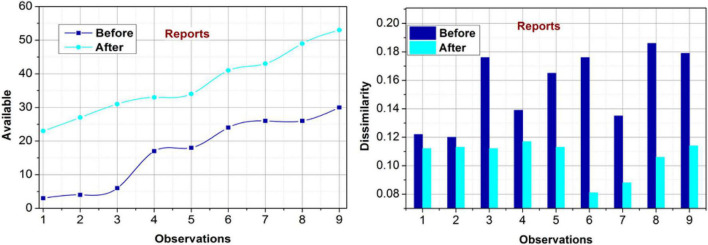
Availability and dissimilarity (reports).

Figure 7 presents the analysis of availability and dissimilarity before and after rule implication using the proposed model. The proposed method provides insights for *M^x^* (*C*) over varying observations for retaining a maximum availability. The unavailable instances are classified asρ(C*S) for improving the accuracy. Based on the decisions from the policies and its players, (*c^g^p*) modifications for the report availability are performed. In particular, the occurrences increase as the relationship between the blocks (classified) increases. Therefore, the dissimilarity between the management blocks and conventional public reports are jointly analyzed throughout the process to improve the*R*_*Im*_. This enhances the availability under different occurrences; the same is pursued between distinguishable features for preventing interruptions and increases in dissimilarity. From the total events to the deviations observed in the data source, the rule implications and policy changes related to the conditions are performed to identify the proposed method’s impact.

#### Impact on sharing

**FIGURE 8:**
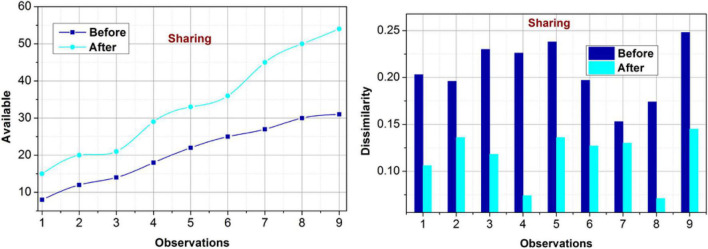
Availability and dissimilarity (sharing).

The availability and dissimilarity under varying observations for the sharing policy are analyzed in Figure 8. The proposed method focuses on the maximum availability of any rule and its impact on the governance policy. This feature is validated using *S^p^* for unclassified occurrences using the transfer learning paradigm. In the further iterations, the additional metrics such as *S*_∃_ and *ch*_*p*_ are analyzed for preventing overlaps in decisions. This ensures mutual consent over the modifications using *C_i_* or *C_p_*. Therefore, the available policy is updated with the modified rule over varying instances. The dissimilarity measure is reduced based on new*R*_*Im*_ as recommended from the*ch*_*p*_∀*i* ∈ *p*. This is repeated untilρ(*S*) is validated for mutual *D^M^*(*c^g^*,*p*) such that the availability is ensured. Besides, the proposed model relies on the previous data from which a concrete solution for sharing policy and its modifications is replicated. Therefore, the dissimilarity measure is reduced over varying observations, improving the availability.

#### Impact on voting

**FIGURE 9:**
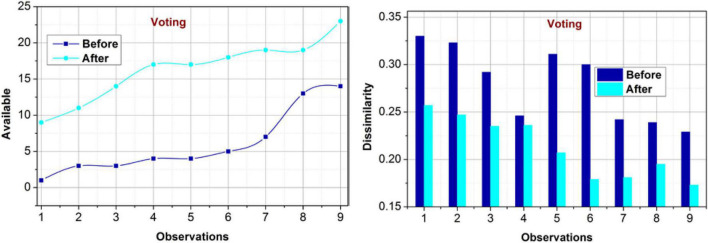
Availability and dissimilarity (voting).

For the varying occurrences, the proposed model achieves better availability for voting policies (Refer to Figure 9). For different players’ input, the proposed model performs classification for matching *M_x_* (*C*) based on *C_i_* and *C_p_*. This requirement is validated using the learning paradigm for delivery policy change and controlled operations. The validation verifies the maximum achievable rules through mutual consent between the consumers, Board, management, and shareholders. Therefore, the diverse considerations are converged throughρ(*S*) that modifies $P_{L}^{R}$ [in Eq. 4] as in Eq. 6. This is performed using*p*, in which maximum policy updates are performed. The performance validations are provided using*R*_*Im*_[ρ(*C*|*S*)] that improves the availability. Different from the other validations, the validations the dissimilarity is identified from the training phase wherein the decision-making is precise over the individual and joint features. Therefore, the policies are updated using further modified*R*_*Im*_.

#### Impact on complaint

**FIGURE 10:**
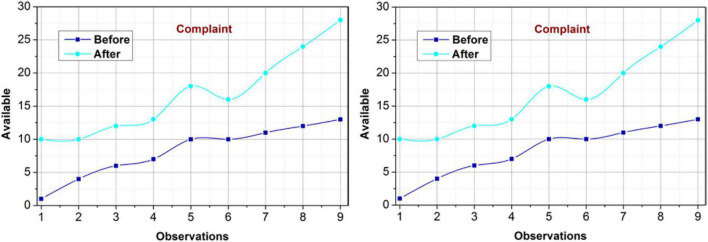
Availability and dissimilarity (complaint).

The proposed model is reliable over the complaint rule availability by reducing the dissimilarities using different metrics in the learning process. The first process is the decision probability analysis using Eq. 7. This comes into existence post the *c^g^* demands such that the*ch_p_* is required. Depending on the changes necessary, the learning verifies(*C*_*i*_,*C*_*p*_,*S*_∃_) under different decisions from the management and relationship blocks. This ensures multiple computations over the varying $P_{L}^{R}$. Initially, it relies on *S_p_* + *S*_∃_ whereas if a dissimilarity is observed, then *M_x_* induced *ch*_*p*_ is performed. In the further data analysis, the proposed model induces 1 − ∃_*ip*_ for improving the availability based on modified rules. The*R*_*Im*_ is administered using different*ch*_*p*_ implications. This is required to prevent dissimilarities between varying input data. Therefore, this aftermath of the proposed model implication maximizes availability and reduces dissimilarity (Refer to Figure 10).

#### Impact on market

**FIGURE 11:**
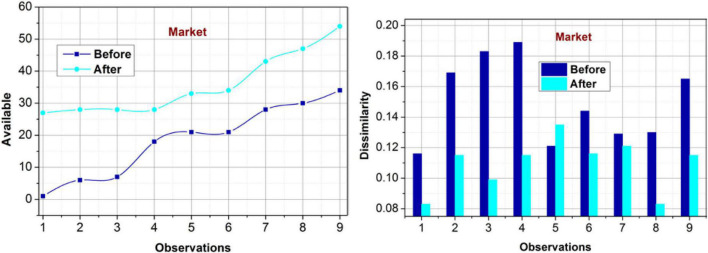
Availability and dissimilarity (market).

The impact of varying occurrences on the market data availability (post*R*_*Im*_) and dissimilarity is analyzed in Figure 11. The proposed model exploits the prime consideration of $\left[\frac{{\left(C\right)}_{i}}{\sum {\left(C+S\right)}_{p}}\right]$ based on the policies between the shareholders and consumers. This relationship generates multiple constraints based on *S_p_* at the initial state. This is violated if the other blocks/management requirements modify the controlled operations. Based on the modified $P_{L}^{R}$, the decisions are performed. The actual management policy is retained under different constraints in the decision-making process. The constraints based on $P_{L}^{-\frac{c\,h}{A}.\exists .i}$ generate new validation requirements. Therefore, further analysis usingρ(*S*) and *S*_∃_ for distinguishable features is analyzed. The training process exploits this analysis under different observations. Therefore, the precise demands are identified for improving the specified delivery between the users and shareholders. This is recurrent until a new query or modification is recommended for *R*_*Im*_. The changes in *C_i_* and *C_p_* jointly incur the governance policies for further assessments. Therefore, the dissimilarities are confined, preventing additional errors and thus improving the availability.

## Conclusion

Artificial intelligent solutions in corporate governance decisions are reliable for meeting the current market/consumer demands. The prime requirement is the data available through protected/interrupted decisions due to different relationships and blocks in corporate governance. Understanding this protective demand, this article introduced a mutual-consent-based governance regulation model for clear governance policy implications. The proposed model exploits transfer learning for distributed feature analysis and decision-making. It aims to reduce dissimilarities in different operational fields due to distinct relationships and governance blocks. Based on the dissimilarities, the existing rules are modified with mutual consent with the blocks preventing unavailability. The decisions are distinct based on previous and current data availability states for easy decision-making. Therefore, impersonated or barged decisions are prevented due to internal relationships between the organizational blocks. With the actual policy formulation, the rule implications based on learning decisions are incorporated to improve policy availability. Therefore, this model is reliable for administering successful corporate governance where dissimilarities between controlled operations are high. As mentioned earlier, the varying data availability instances will be analyzed using this model from mediate to maximum decision-making over rule implications. This process requires data normalization for balancing unavailability and availability features over different governance policies. In future, solid financial decisions are required to improve corporate governance with minimum risk conditions. Therefore, risk management committees must be appointed to improve company performance.

## Data availability statement

The original contributions presented in this study are included in the article/supplementary material, further inquiries can be directed to the corresponding author.

## Author contributions

The author confirms being the sole contributor of this work and has approved it for publication.
